# Supplementary material for: Prefrontal cortical activation measured by fNIRS during walking: effects of age, disease and secondary task
Source: PeerJ. 2019 May 3;7:e6833. doi: 10.7717/peerj.6833 (PMC6501770; doi:10.7717/peerj.6833)
Supplement: Supplemental Information 2 [file peerj-07-6833-s002.docx]

Several reports of brain activation during walking using functional near-infrared spectroscopy (fNIRS) have been published in the last decade. Activation of the Prefrontal Cortex (PFC) (easily accessible using fNIRS) has often been investigated during walking tasks. Brain motor areas investigated also include the Pre Motor Cortex, the Pre Supplementary Motor Area, the Supplementary Motor Area and the Sensory Motor Cortex. Thus, there is now considerable literature that requires synthesising and systematic review of the main findings related to the brain activation as assessed by fNIRS during walking tasks.

Some recent reviews have examined fNIRS and gait. These reviews have addressed (i) methodological aspects; (ii) data processing techniques; (iii) or restricted their focus to ageing, Parkinson’s disease, Parkinsonism syndromes and Stroke.

Further analysis and synthesis of published fNIRS studies are required to gain a better understanding of (i) brain activation changes during complex walking compared to walking or standing; (ii) brain activation patterns in young healthy people as this group provides the model of intact cognitive functioning; and (iii) brain functioning in diverse clinical groups with walking and neurological impairments. A methodological scale is also required to assist in the evaluation of the literature published to date.

Thus, through this systematic review we summarized the published findings regarding brain activation patterns (primarily focused on PFC activation) during simple and complex walking tasks in young adults, older adults and clinical groups with balance disorders, to gain an insight into neural processes required for ambulation.
